# Supplementary material for: Inhibitory gating of coincidence-dependent sensory binding in secondary auditory cortex
Source: Nat Commun. 2021 Jul 29;12:4610. doi: 10.1038/s41467-021-24758-6 (PMC8322099; doi:10.1038/s41467-021-24758-6)
Supplement: Supplementary file 1 — Supplementary Information [file 41467_2021_24758_MOESM1_ESM.docx]

**
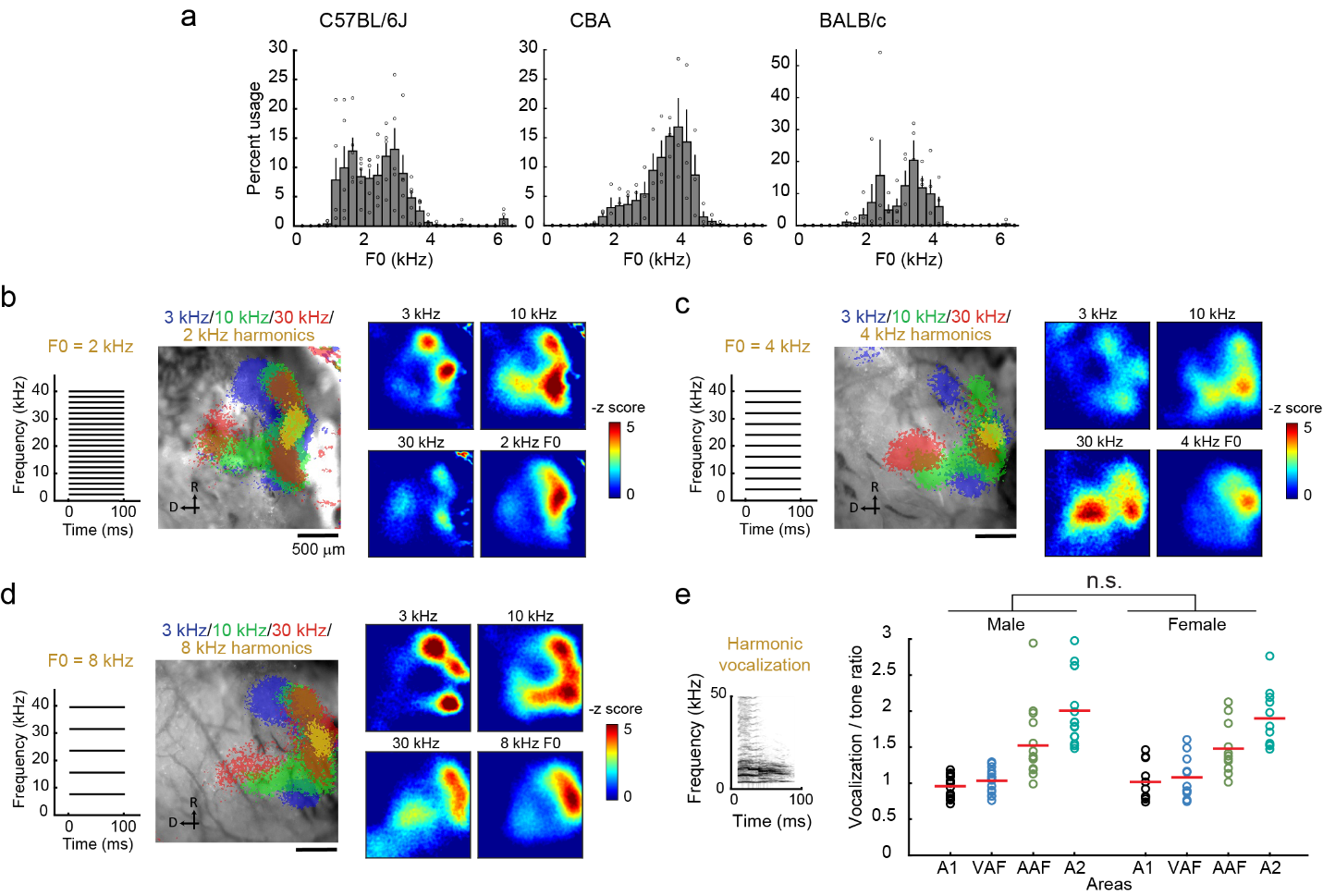
**

**Supplementary Figure 1. Additional analyses for mapping harmonic responses.**

**(a)** Histograms showing the usage probability of fundamental frequency (F0) for harmonic vocalizations in three strains—left, B6 (n = 5 mice, median: 2.5 kHz, 80% of F0s fell between 1.5 and 3.3 kHz); middle, CBA (n = 3 mice, median: 3.5 kHz, 80% fell between 2.1 and 4.3 kHz); right, BALB/c (n = 4 mice, median: 3.3 kHz, 80% fell between 2.6 and 3.9 kHz). Results are mean ± SEM, overlaid with individual data points. **(b)** Left, spectrogram of artificial 2 kHz-F0 harmonics. Middle, thresholded intrinsic imaging signal responses to pure tones as well as harmonics in a representative mouse. Right, heat maps showing z-scored response amplitudes. **(c)** Data for 4 kHz-F0 harmonics. **(d)** Data for 8 kHz-F0 harmonics. **(e)** Ratio of harmonic vocalization to pure tone response amplitudes in each of the auditory cortical areas for male and female mice. Left, males (n = 15 mice); right, females (n = 11 mice). No difference was observed between males and females (male vs female, p = 0.947; areas, p = 5.04×10^-17^; interaction, p = 0.813; Two-way ANOVA). Red lines show mean.


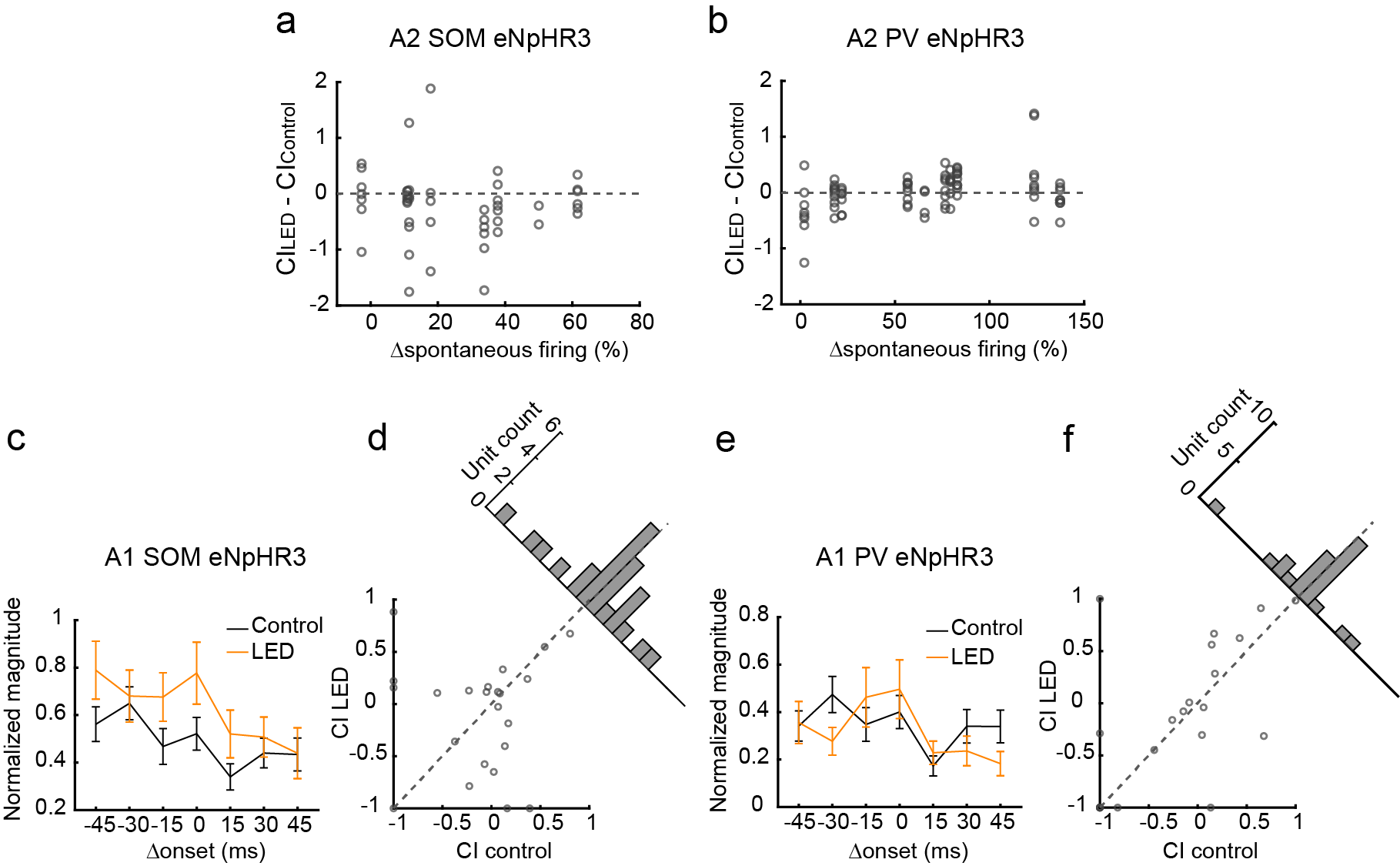


**Supplementary Figure 2. Optogenetic inactivation of inhibitory neurons does not change coincidence preference in A1.**

**(a)** Scatter plot showing change in CI against spontaneous firing rate change triggered by SOM cell photoinactivation in individual regular-spiking units (n = 6 mice, 47 units, allowing duplication of the same units across two photostimulation intensities). Units with spontaneous firing rate less than 0.25 Hz were excluded. **(b)** The same plot for PV cell photoinactivation (n = 7 mice, 81 units). **(c)** Summary plot showing the response amplitudes of A1 regular-spiking units to 4 kHz-F0 harmonics with various Δonsets during control and SOM cell inactivation trials. Responses are normalized to the maximum response amplitude in the control condition in each unit and averaged across all units (n = 5 mice, 24 units in the A1 superficial layer). **(d)** Scatter plot showing CI during control and SOM cell inactivation trials. The oblique histogram illustrates the changes in CI with LED. p = 0.913 (two-sided paired t-test). **(e)** Summary plot showing the response amplitudes of A1 regular-spiking units during control and PV cell inactivation trials (n = 6 mice, 25 units in the A1 superficial layer). **(f)** Scatter plot showing CI during control and PV cell inactivation trials. The oblique histogram illustrates the changes in CI with LED. p = 0.551 (two-sided paired t-test). Results show mean ± SEM in (c) and (e).

**
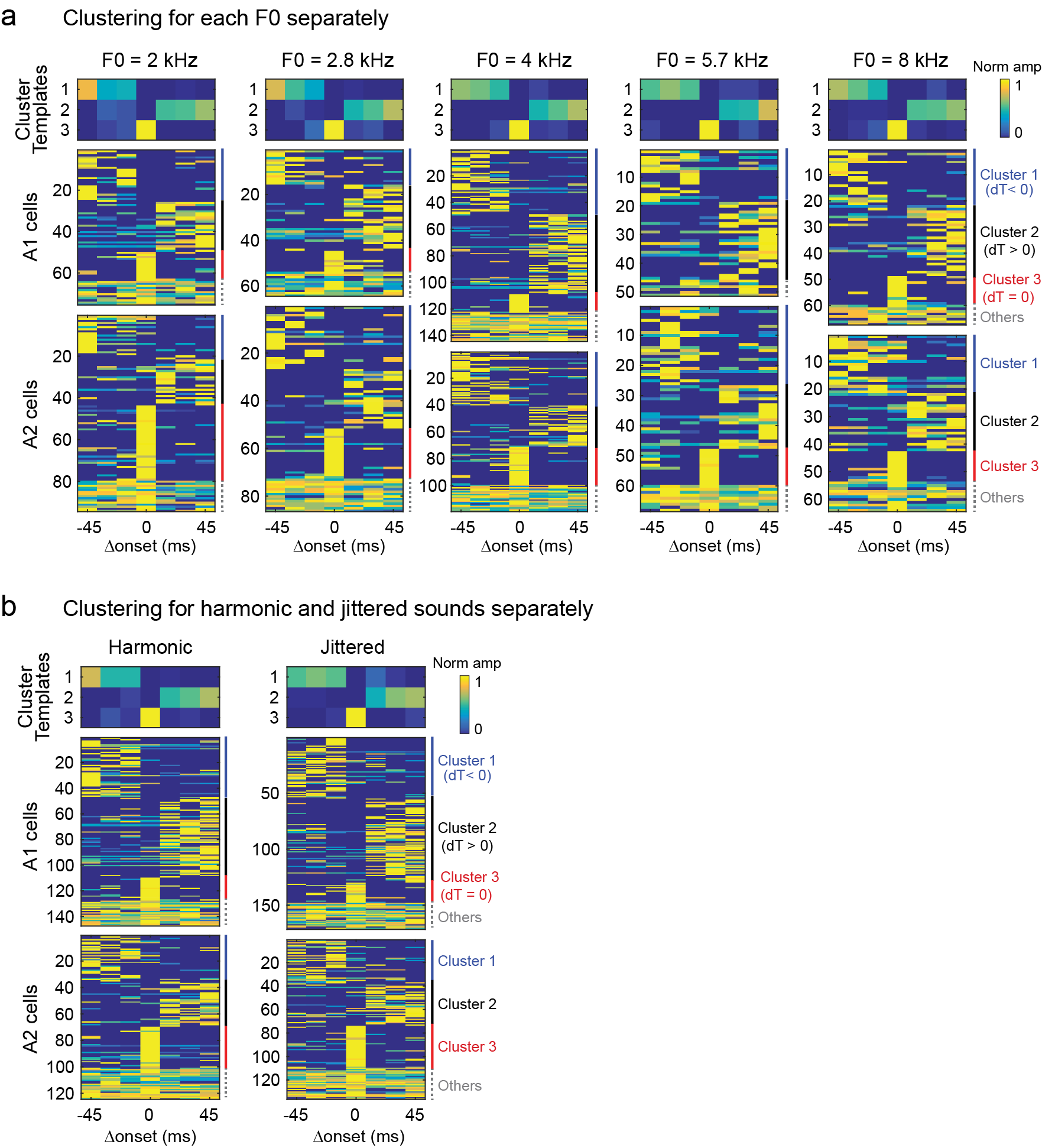
**

**Supplementary Figure 3. Reproducible clustering of A1 and A2 neurons into negative shift-, positive shift-, and coincidence-preferring groups for individual F0s and jitters.**

**(a)** Clustering data independently conducted for each F0. The order of appearance of three clusters was matched to that in Figures 5 and 6. F0 = 2 kHz (A1: n = 75 cells, A2: n = 94 cells); F0 = 2.8 kHz (A1: n = 64 cells, A2: n = 86 cells); F0 = 4 kHz (A1: n = 147 cells, A2: n = 119 cells); F0 = 5.7 kHz (A1: n = 51 cells, A2: n = 68 cells); F0 = 8 kHz (A1: n = 67 cells, A2: n = 64 cells). Clustering was robust even when conducted separately for individual F0s. **(b)** Clustering data independently conducted for harmonic and jittered sounds, Harmonic (A1: n = 147 cells, A2: n = 124 cells); Jittered (A1: n = 171 cells, A2: n = 136 cells).

**
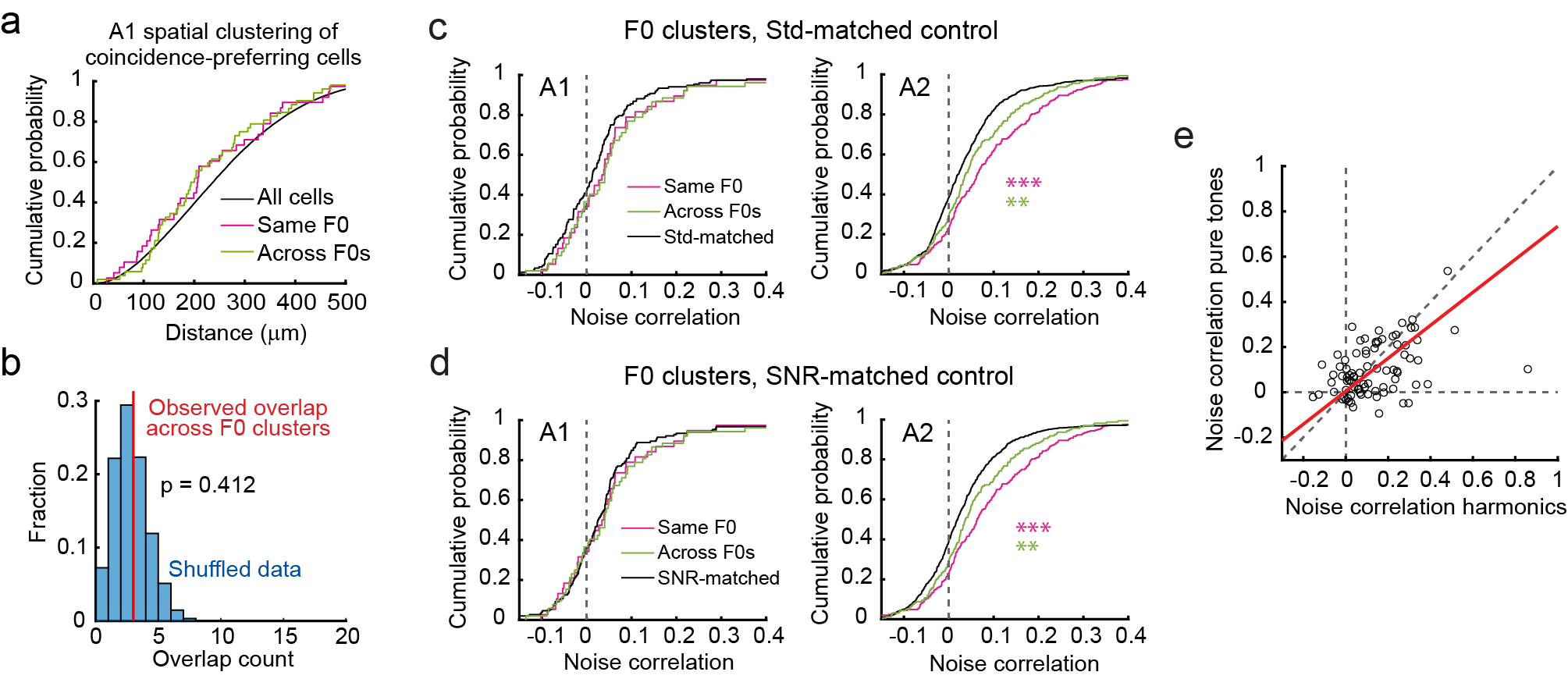
**

**Supplementary Figure 4. Additional data for clustering analyses of harmonics-responding neurons across F0s.**

**(a)** Cumulative probability plot of spatial distance between all cells (black), between coincidence-preferring cells with the same F0 (magenta), and between coincidence-preferring cells across F0s (green) in A1 (n = 46662, 38, 52 for All cells, Same F0, and Across F0s). **(b)** Count of overlap between coincidence-preferring cells across F0s for A1. Red line, observed overlap count; histogram, distribution of overlap count for shuffled data (10,000 repetitions, p = 0.412, permutation test). **(c)** Cumulative probability plots of noise correlation between coincidence-preferring cell pairs along with standard deviation (Std)-matched control (A1: n = 38, 52, 152; A2: n = 248, 159, 992 for Same F0, Across F0s, and Std-matched). p*** < 0.0001, p** < 0.01. **(d)** Cumulative probability plots of noise correlation between coincidence-preferring cell pairs along with signal-to-noise ratio (SNR)-matched control. Two-sided Wilcoxon rank sum test for (a), (c), and (d). **(e)** Noise correlation was measured for identical Same-F0 cell pairs across two days (harmonics and pure tone experiments) and plotted against each other. We observed a positive correlation across days (n = 84 cell pairs, R = 0.421, p = 8.33×10^-5^, two-sided t-test). Out of all cell pairs, 68.3% fell within the upper-right quadrant, indicating positive noise correlation on both days. Red line indicates a geometric mean regression line. Dotted lines indicate a unity line and quadrant boundaries. See Supplementary Data 1 for additional statistics.

**
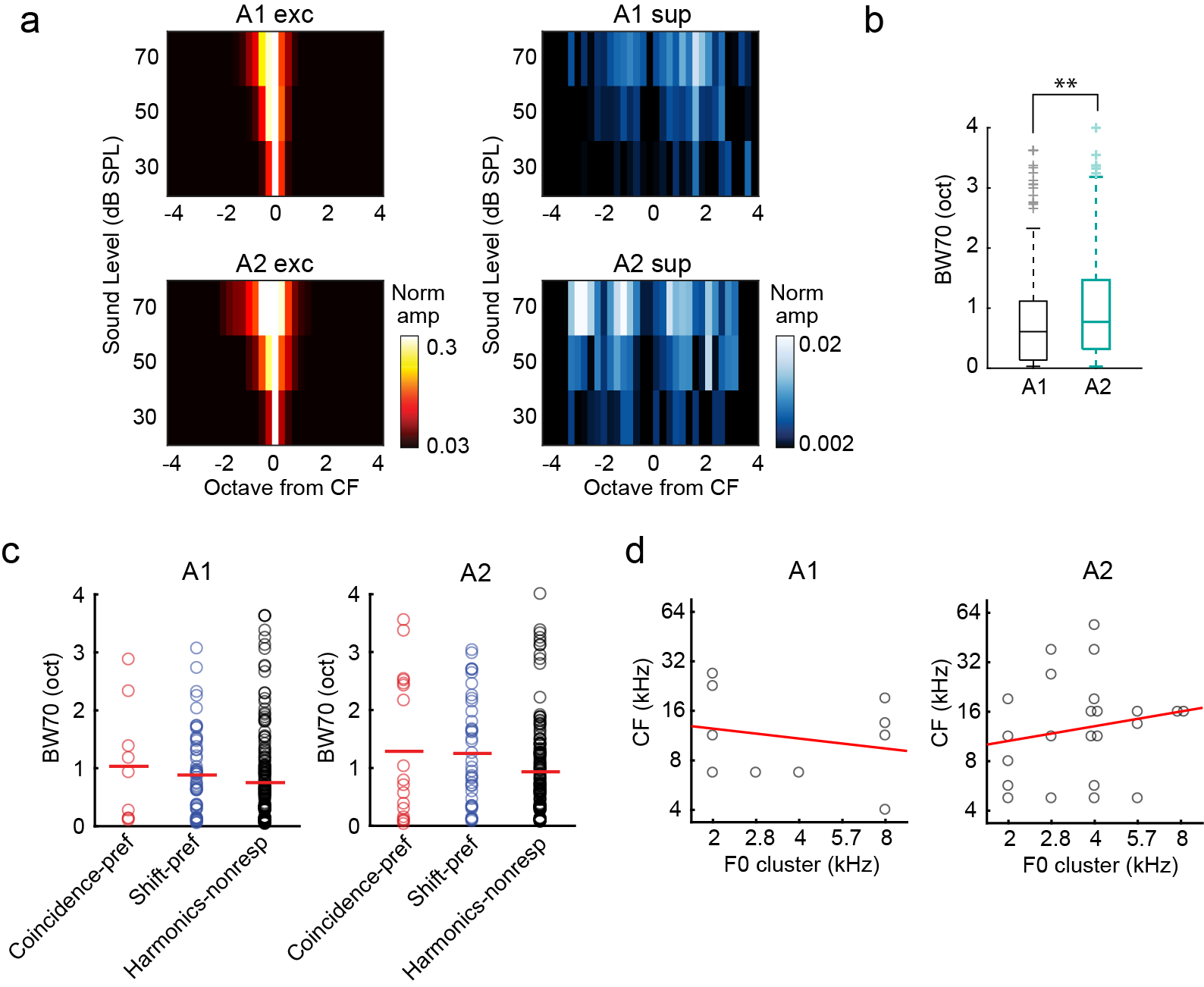
**

**Supplementary Figure 5. Tonal receptive fields of A1 and A2 neurons.**

**(a)** Tonal receptive fields of excitatory and suppressive responses to pure tones averaged across all cells with excitatory responses in A1 (n = 296 cells, 11 mice) and A2 (n = 232 cells, 10 mice). Responses are centered around the characteristic frequency (CF) of excitatory response for each cell. **(b)** Tuning broadness of neurons in each area measured as bandwidth at 70 dB SPL (BW70). Box plots show median and 25th and 75th percentiles as box edges, and 1.5 × interquartile range as whiskers. Crosses indicate outliers. **p = 0.0093 (two-sided t-test). **(c)** Bandwidth at 70 dB SPL (BW70) separately quantified for individual groups based on Δonset-dependent harmonics response types. The ‘Coincidence-pref’ group includes all neurons which are judged as coincidence-preferring cluster in any of five F0s. ‘Shift-pref’ group includes all neurons which are judged as positive Δonset-preferring or negative Δonset-preferring cluster, but not coincidence-preferring cluster in any of five F0s. The ‘Harmonics-nonresp’ group includes neurons which were responsive to pure tones but not responsive or unmeasured for harmonic sounds (A1: Coincidence-pref, n = 9 cells, Shift-pref, n = 58 cells, Harmonics-nonresp, n = 176 cells, p = 0.361. A2: Coincidence-pref, n = 18 cells, Shift-pref, n = 41 cells, Harmonics-nonresp, n = 149 cells. p = 0.0546; One-way ANOVA. No significant difference was found between groups). Red lines show mean. **(d)** Characteristic frequency of coincidence-preferring neurons for each F0 in A1 and A2 (A1: slope = -0.216, p = 0.549, n = 10 cells responsive to pure tones out of 26 cells which were classified as coincidence-preferring; A2: slope = 0.178, p = 0.418, n = 23 out of 69 cells, two-sided t-test). We did not observe a relationship between preferred F0 and characteristic frequency of coincident harmonics-preferring neurons, suggesting that these neurons do not encode pitch. Red lines show linear regression. See Supplementary Data 1 for additional statistics.

**
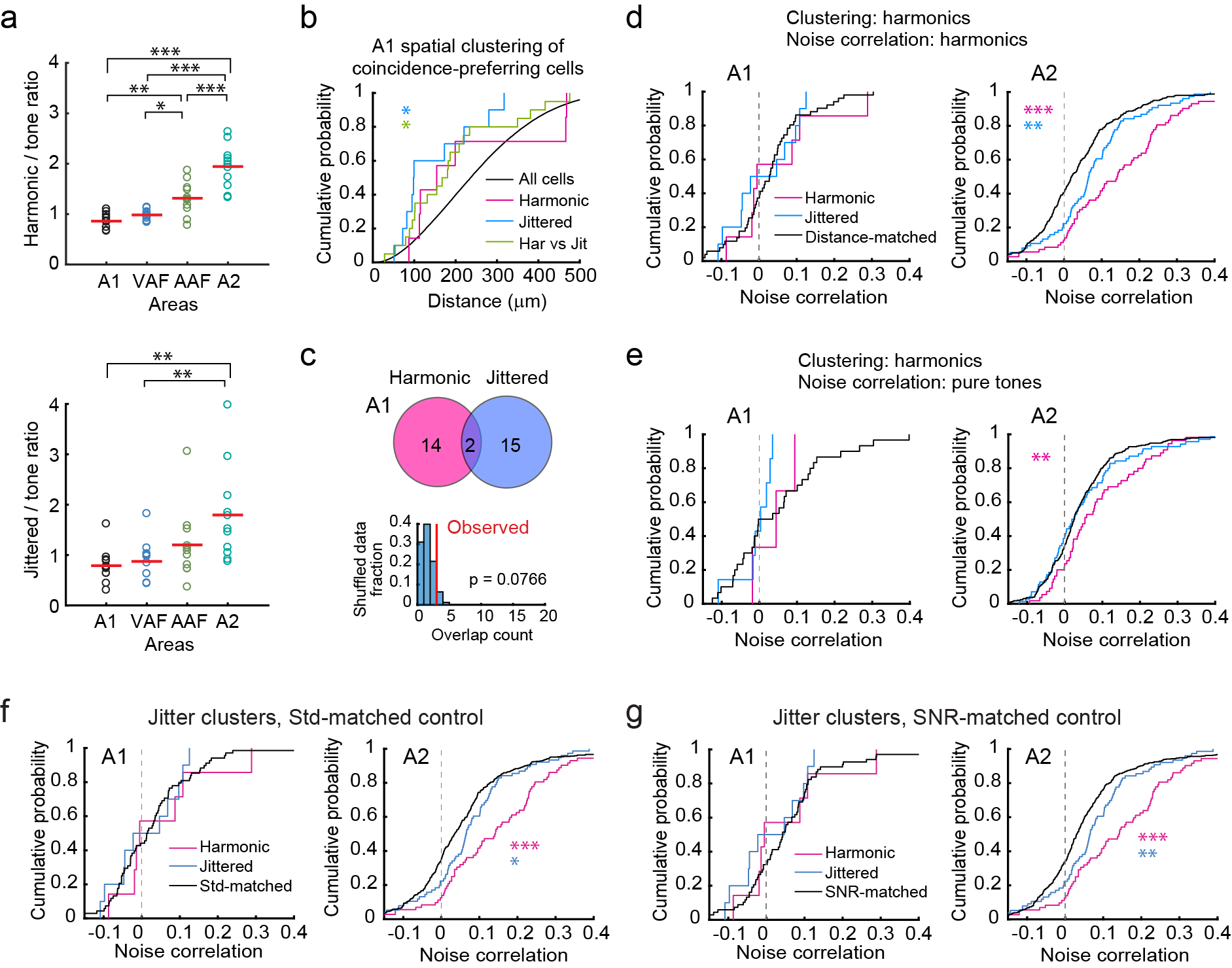
**

**Supplementary Figure 6. Additional data for clustering analyses of jittered harmonics-responding neurons.**

**(a)** Top, ratio of 4 kHz-F0 harmonics to pure tone response amplitudes in each auditory cortical area (n = 11 mice, p*** < 0.001, p** < 0.01, p* < 0.05 (One-way ANOVA followed by Tukey’s HSD test). Bottom, ratio of jittered harmonics to pure tone response amplitudes in the same mouse. **(b)** Cumulative probability plot of spatial distance in A1 between all cells (black), between coincidence-preferring cells for harmonics (magenta), between coincidence-preferring cells for jittered sounds (blue), and between coincidence-preferring cells for harmonic and jittered sounds (green) (n = 44610, 7, 10, 20 for All cells, Harmonic, Jittered, and Har vs Jit). *p < 0.05. **(c)** Top, Venn diagram showing the overlap between coincidence-preferring clusters for harmonic and jittered sounds in A1. Bottom, observed overlap of coincidence-preferring cells for harmonics and jittered sounds (red line) compared to shuffled data (histogram, 10,000 repetitions, permutation test). **(d)** Cumulative probability plots of noise correlation between coincidence-preferring cells for harmonic and jittered sounds in A1 and A2. Data for A2 is the same as Figure 6j (A1: n = 7, 10, 51; A2: n = 72, 76, 399 for Harmonic, Jittered, and Distance-matched). ***p < 0.0001, **p < 0.01 **(e)** Cumulative probability plots of noise correlation between coincidence-preferring cell pairs during presentation of pure tones (A1: n = 3, 7, 30; A2: n = 55, 70, 330 for Harmonic, Jittered, and Distance-matched). **(f)** Cumulative probability plots of noise correlation between coincidence-preferring cell pairs along with standard deviation (Std)-matched control (black) (A1: n = 7, 10, 68; A2: n = 72, 76, 532 for Harmonic, Jittered, and Std-matched). **(g)** Cumulative probability plots of noise correlation between coincidence-preferring cell pairs along with signal-to-noise ratio (SNR)-matched control (black). Two-sided Wilcoxon rank sum test for (b), (d), (e), (f), and (g). See Supplementary Data 1 for additional statistics.

**
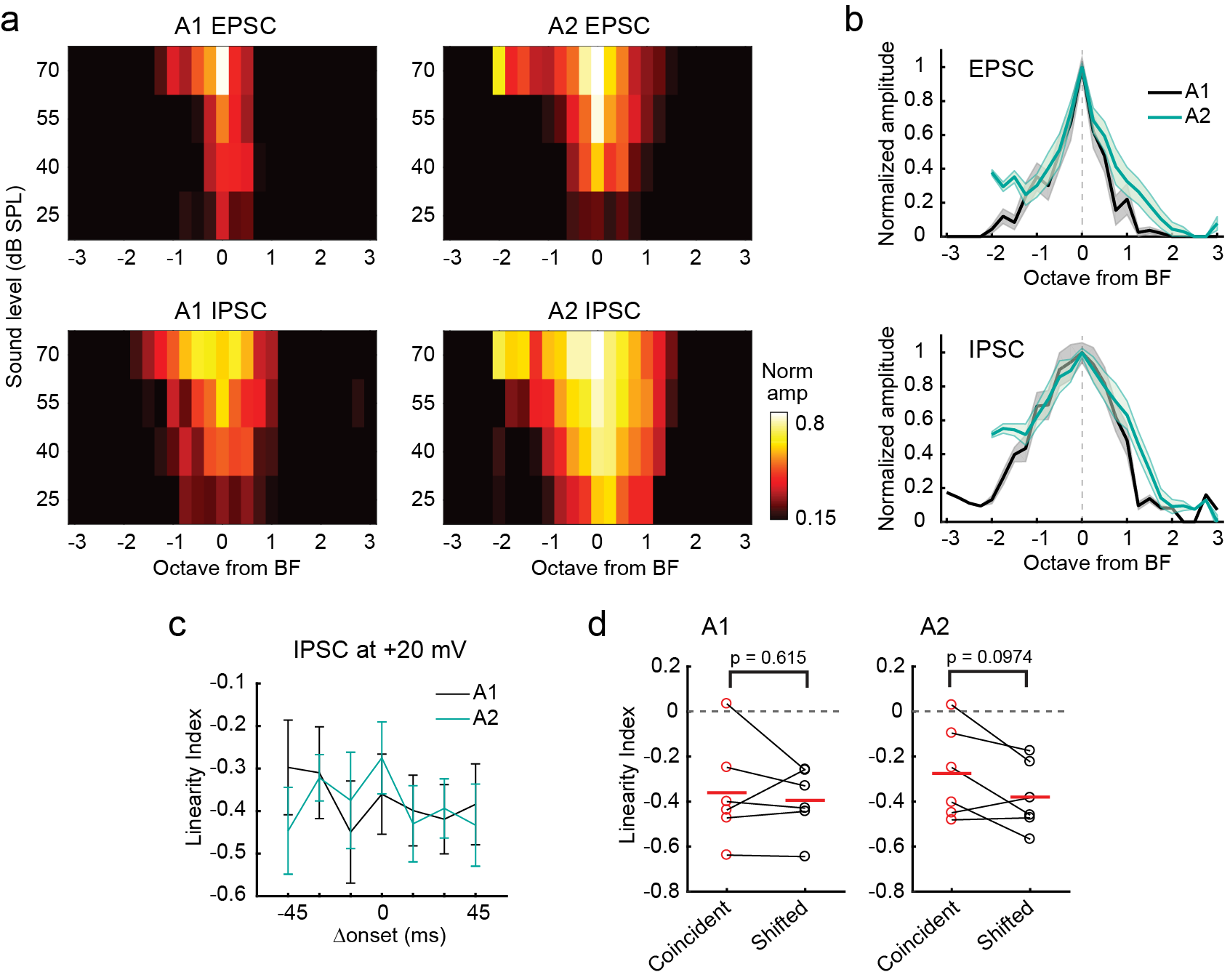
**

**Supplementary Figure 7. Tonal receptive fields and three-tone harmonics responses of synaptic currents in A1 and A2.**

**(a)** Tonal receptive fields of EPSCs and IPSCs in response to pure tones averaged across cells in A1 (EPSCs: n = 16 cells, IPSCs: n = 16 cells) and A2 (EPSCs: n = 6 cells, IPSCs: n = 6 cells). Responses are centered around the best frequency of excitation for each cell. **(b)** Summary of EPSC and IPSC frequency tuning in A1 and A2. Response amplitudes are normalized to their individual peaks. Dark line, mean; shading, SEM. **(c)** Linearity index of IPSCs calculated for each Δonset in A1 and A2 (A1: n = 6; A2: n = 6 cells). Results show mean ± SEM. **(d)** Summary plots showing the linearity index of IPSCs calculated for coincident and shifted harmonics (A1: n = 6 cells, p = 0.615; A2: n = 6, p = 0.0974, two-sided paired t-test). Red lines show mean.


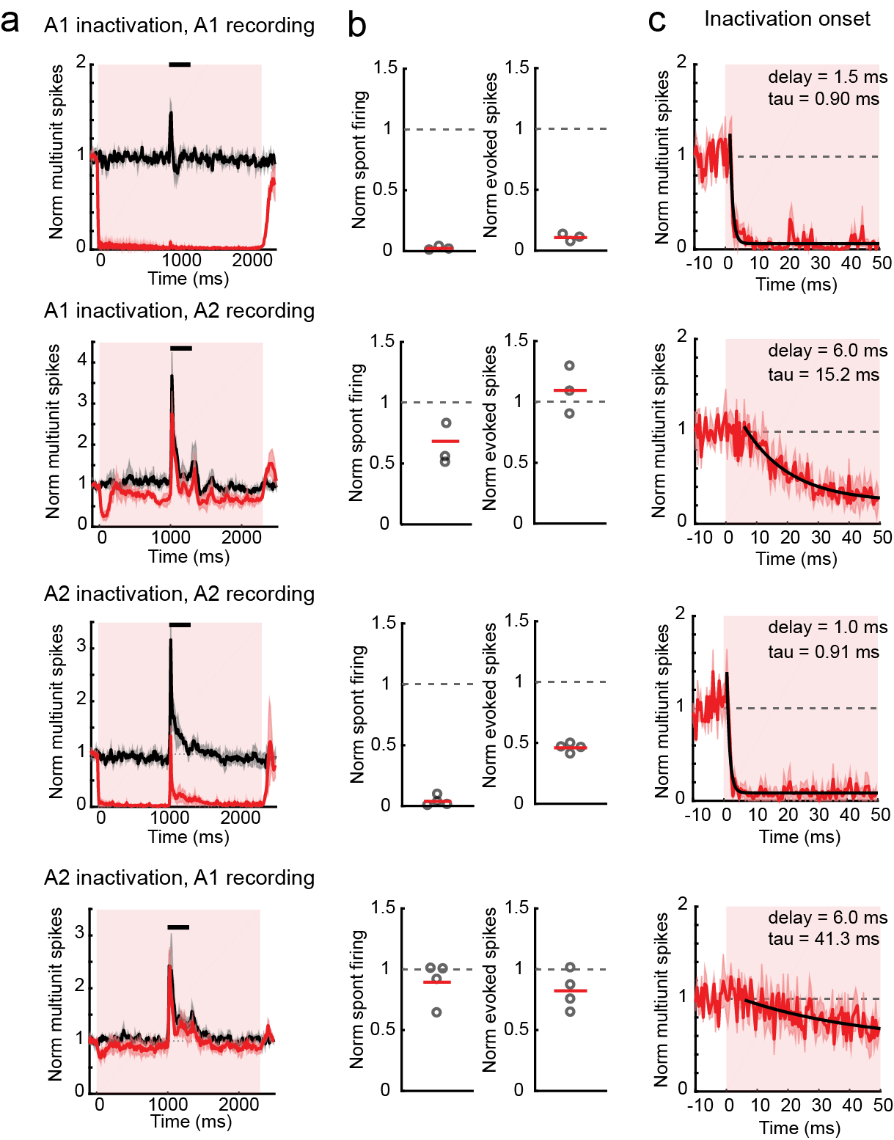


**Supplementary Figure 8. Optogenetic inactivation is restricted to the targeted areas.**

**(a)** Functional inactivation of cortical areas across recording conditions: A1 inactivation, A1 recording (n = 3 mice); A1 inactivation, A2 recording (n = 3); A2 inactivation, A2 recording (n = 4); A2 inactivation, A1 recording (n = 4). PSTHs show multiunit spikes during control (black) and photostimulation (red) trials. **(b)** Summary results showing the reduction of spontaneous and evoked firing rate during photostimulation. A1 inactivation, A1 recording (n = 3 mice); A1 inactivation, A2 recording (n = 3); A2 inactivation, A2 recording (n = 4); A2 inactivation, A1 recording (n = 4). Red lines show mean. **(c)** Multiunit spikes during the first 50 ms of photostimulation showing inactivation kinetics rapidly after LED onset. Black lines, single-exponential fit. A1 inactivation, A1 recording and A2 inactivation, A2 recording data display rapid decay time constants with < 1 ms latency, indicating their direct inactivation. In contrast, A1 inactivation, A2 recording and A2 inactivation, A1 recording data show only slow decay with long latency, confirming the lack of direct photoinactivation. Solid traces and shades show mean ± SEM in (a) and (c).

**
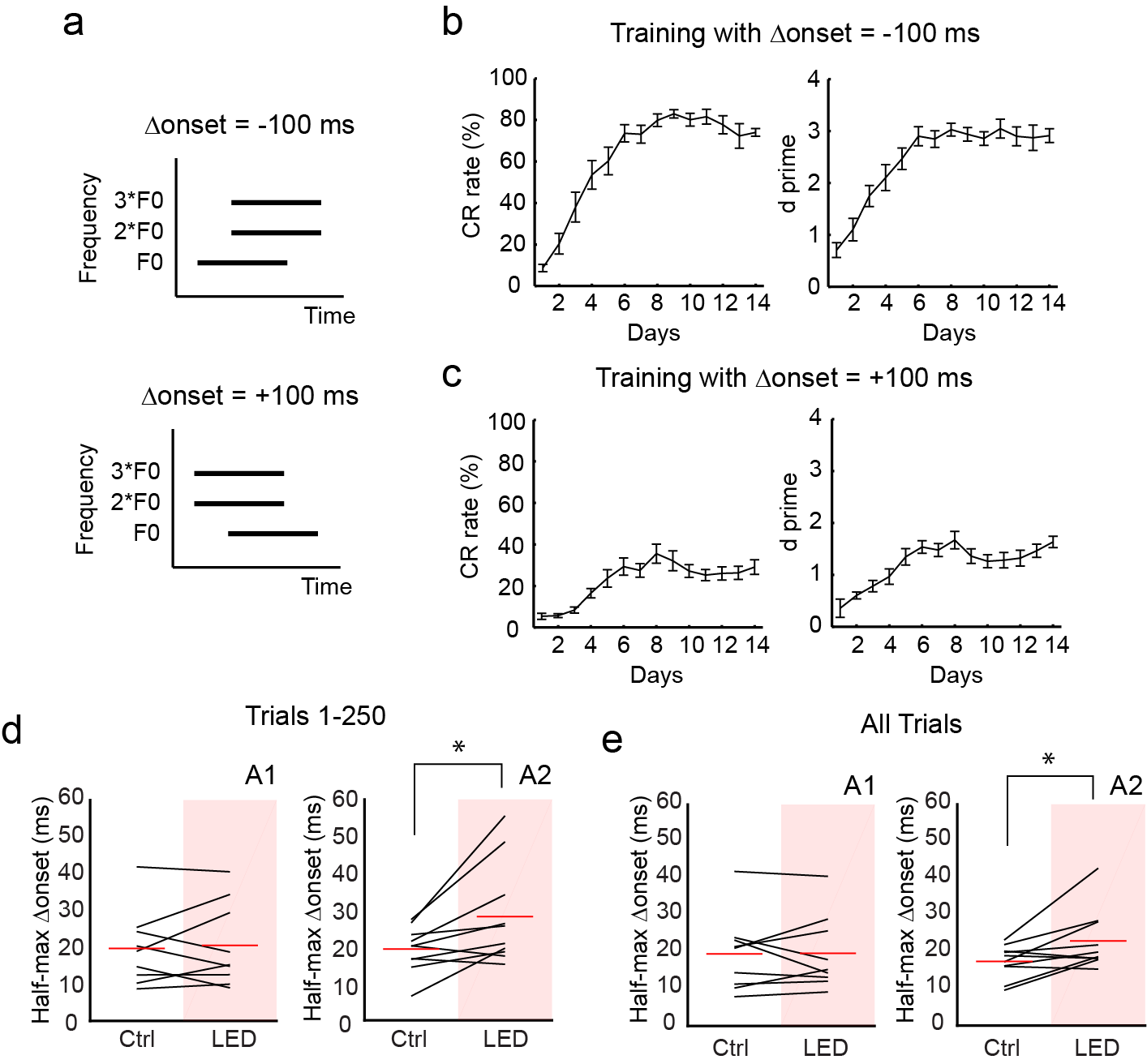
**

**Supplementary Figure 9. Harmonics discrimination training with negative and positive onset shifts.**

**(a)** Schematic for three-tone harmonic stimulus with -100 ms and +100 ms Δonsets. **(b)** Learning curves averaged across all tested mice (n = 19) during training with Δonset of -100 ms. Left, average correct rejection rate over days. Right, average d prime over days. Results are mean ±SEM. (The same data as Fig. 8c) **(c)** Same as (b) except for Δonset of +100 ms. **(d)** Half-max Δonsets with and without inactivation of A1 (left) or A2 (right), using the first 250 trials (A1: n = 9; A2: n = 10 mice; the same data as Fig. 8j). **(e)** The same data using all trials in each mouse (486 +/- 31 trials). A1 control: 19.5 ± 3.4, LED: 19.7 ± 3.4; A2 control: 17.4 ± 1.4, LED: 23.0 ± 2.6. *p = 0.0273 (two-sided Wilcoxon signed rank test).


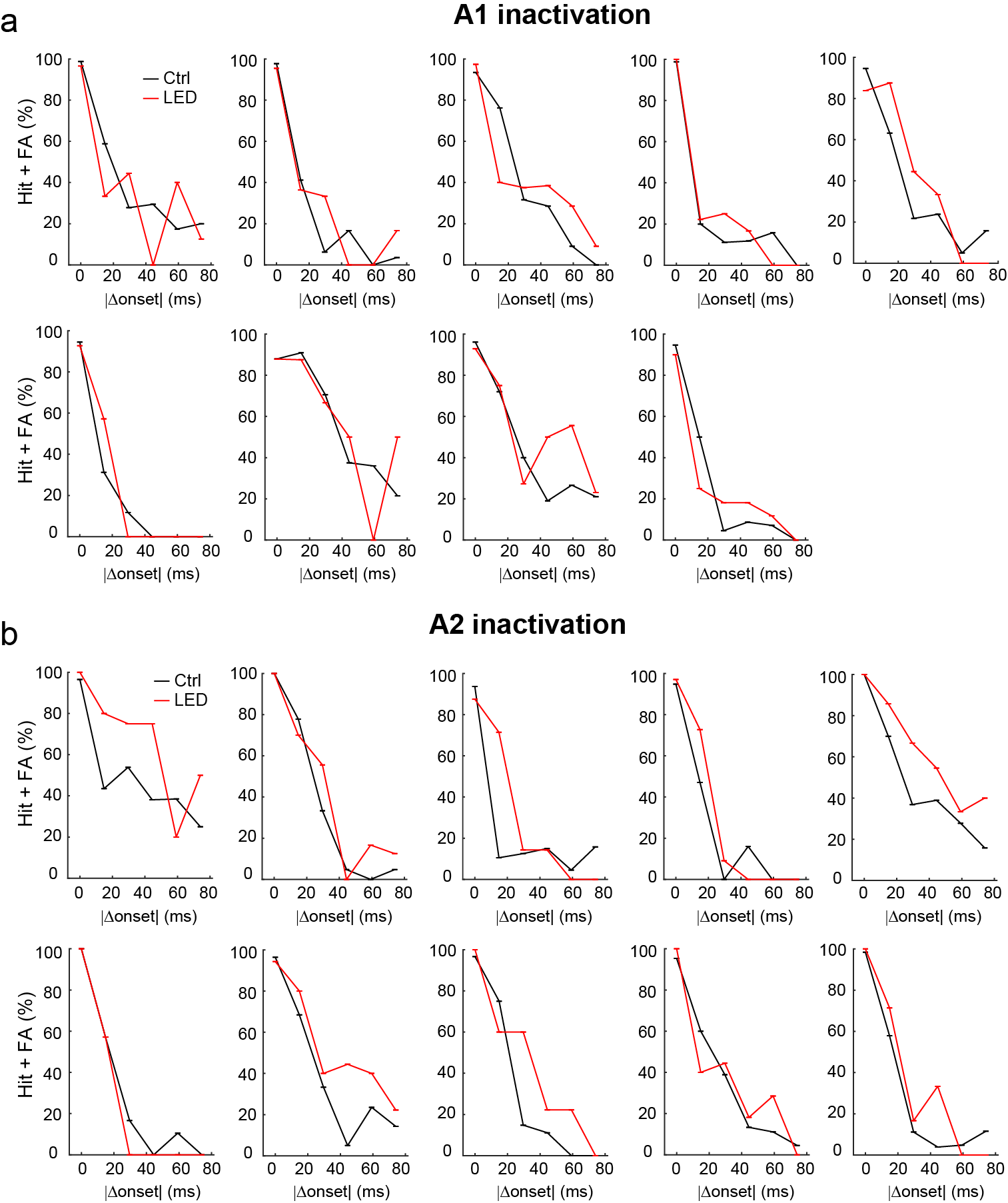


**Supplementary Figure 10. Data for all mice in the harmonics discrimination task.**

**(a)** Discrimination psychometric curves against Δonset with (black) and without (red) optogenetic inactivation of A1 in nine mice. **(b)** Discrimination psychometric curves against Δonset with (black) and without (red) optogenetic inactivation of A2 in ten mice.
