# Supplementary material for: Inhibitory gating of coincidence-dependent sensory binding in secondary auditory cortex
Source: Nat Commun. 2021 Jul 29;12:4610. doi: 10.1038/s41467-021-24758-6 (PMC8322099; doi:10.1038/s41467-021-24758-6)
Supplement: Supplementary file 3 — Description of Additional Supplementary Files [file 41467_2021_24758_MOESM3_ESM.pdf]

Description of Additional Supplementary Files

Supplementary Data 1: Summary of statistics used in all figures.
